# Study on the Effect of Recycled Fine Aggregate Qualities on Fly Ash/GGBS-Based Geopolymer Mortar

**DOI:** 10.3390/ma16237289

**Published:** 2023-11-23

**Authors:** Shilun Liu, Zihao Liu, Koji Takasu, Hidehiro Koyamada, Hiroki Suyama

**Affiliations:** 1 Architecture Course, Graduate School of Environmental Engineering, The University of Kitakyushu, 1-1 Hibikino Wakamatsu, Kitakyushu 8080135, Fukuoka, Japan; c1dbb422@eng.kitakyu-u.ac.jp (S.L.); z-liu@kitakyu-u.ac.jp (Z.L.); 2 Department of Architecture, Faculty of Environmental Engineering, The University of Kitakyushu, 1-1 Hibikino Wakamatsu, Kitakyushu 8080135, Fukuoka, Japan; h-koyamada@kitakyu-u.ac.jp (H.K.); suyama@kitakyu-u.ac.jp (H.S.)

**Keywords:** recycled fine aggregate, geopolymer mortar, compressive strength, drying shrinkage, microstructure, RFA preprocessing method

## Abstract

The rapid expansion of construction, fueled by industry and economic and population growth, has exacerbated the challenge of managing construction waste, especially concrete waste. One promising solution lies in the utilization of recycled fine aggregate (RFA), especially in combination with the emerging geopolymer technology, an innovative alternative to traditional cement. This study systematically explores the effects of incorporating varying qualities and quantities of RFA into geopolymer mortars. By using GGBS and FA as raw materials and replacing natural aggregates (NA) with RFA at different rates (25%, 50%, 75%, and 100%), the research investigates the fresh properties, mechanical characteristics, and drying shrinkage of geopolymer mortar. Key findings reveal that RFA significantly influences the flowability of geopolymer mortar: when RFA content is above 75%, preprocessed RFA (with particles below 0.15 mm removed) has substantially improved flowability, increasing it more than 20%. The critical impact of RFA preprocessing on enhancing mechanical properties and the higher the inclusion level (above 75%), the more pronounced is the advantage in enhancing the compressive strength compared to unprocessed RFA. Additionally, RFA was found to contribute to a denser interfacial transition zone (ITZ) than natural aggregate, which helps maintain the compressive strength at increased RFA dosages. Contrary to findings in cement mortar, a positive correlation exists between pore volume and compressive strength in geopolymer mortar incorporating RFA. This study underscores the potential of refined RFA preprocessing methods in advancing sustainable construction, highlighting avenues for the broader application of RFA in geopolymer mortar.

## 1. Introduction

Economic and population growth in recent years has stimulated the construction industry to flourish and raised the demand for traditional building materials [1]. Natural aggregate (NA) consumption reached 50 billion tons annually in 2020, and is projected to increase to 60 billion tons between 2030 and 2050 [2]. This high demand for natural aggregates puts a lot of stress on the surrounding ecosystems [3,4]. Meanwhile, the construction industry’s development has led to a growing amount of construction waste yearly, with concrete waste making up the largest share [5]. Construction and demolition waste (C&D waste) accounts for about one-third of the materials in US landfills [6]. The amount of C&D waste in 2014 was about 1.13 billion tons in China [7] and more than 4500 million tons in Brazil [8]. C&D waste is generated in large quantities and the severe shortage of raw mineral resources [9] is an environmental problem that must be faced now. Recycled fine aggregate (RFA) can help solve this environmental problem effectively. RFA from C&D waste is a composite materials made of cement, aggregates, water, and final admixtures or materials that partially replace cement [4]. The composition of RFA shows that it possesses the potential to replace natural sand. Therefore, using RFA in the construction industry will alleviate the environmental problems caused by the overuse of NA [10].

The research and application of geopolymers to replace cement are also advancing steadily. The development of geopolymers offers promising ways to convert industrial waste into resources [11,12]. Applying geopolymers will minimize pollution, especially carbon dioxide emissions [13]. Compared with Portland cement, geopolymers have superior mechanical properties (such as higher compressive strength [14], higher tensile strength [15]), and durability properties (such as lower drying shrinkage [16] and stronger fire resistance [17]).

Incorporating RFA into geopolymer mortar can have a significant impact on the construction industry’s sustainable development; however, the results of many studies show that RFA’s drawbacks are obvious. RFA can reduce the mechanical properties of mortar. Dapana [18] investigated the effect of RFA content on mortar density and compressive and flexural strength properties by using 0%, 5%, 10%, 15%, 20%, and 50% RFA content. The results indicate that using more than 20% RFA reduces the compressive and flexural strength of the mortar. Zengfeng Zhao [19] explored the effect of the ratio and particle type of saturated RFA on the mechanical properties of mortar. The results revealed that the compressive strength of the mortar decreased almost linearly with the increase in the RFA substitution rate. As regards durability, using RFA increases the drying shrinkage and water absorption of the mortar [20,21]. Observing the above findings, the use of RFA may reduce the mechanical properties and durability of mortar, which is not conducive to the promotion and application of RFA in geopolymer mortars.

Some scholars have proposed methods to improve the quality of RFA and recycled coarse aggregate (RCA). These methods include removing the attached mortar by ultrasonic cleaning [22], using microbial-induced mineralization deposition to improve the quality of the RFA [23], heating the RCA and then rubbing it [24], or pre-soaking the RCA with HCl, H_2_SO_4_, and H_3_PO_4_ [25]. However, these methods can negatively impact the materials and the environment [26]. Additionally, some of these processing methods are cumbersome and expensive, making them unsuitable for widespread use.

Li et al. [27] investigated the effect of RFA with different particle size ranges on the rheological behavior and compressive strength of concrete by means of removing particles larger than 2.36 mm and smaller than 0.15 mm from the RFA. The results showed that the use of RFA with the removal of particles of up to 0.15 mm was beneficial to the compressive strength of concrete.

In summary, the method of removing small particles from RFA is the most reproducible and the most suitable for replication, so it can be attempted to be applied to geopolymer mortar to explore its feasibility.

Few studies have been completed on recycled aggregate geopolymer by researchers to date. Saba et al. [28] studied the effect of RFA on the hardened properties of geopolymer mortar based on metakaolin. The results indicated that using more than 40% RFA reduced the strength properties, and mixtures with RFA needed a higher liquid content, which could increase porosity and weaken the bond between the RFA and paste.

Hasnaoui et al. [29] studied geopolymer concrete’s hardened properties when recycled fine and coarse aggregates were used. They found that when the substitution ratio exceeded 10%, the compressive strengths decreased by 18% and 27%, respectively, with 30% and 50% recycled aggregate incorporation. Lyu et al. [30] used waste bricks as RFA to make geopolymer mortar and compared it with river sand aggregate. Their experiment showed that using RFA reduced the strength of geopolymer mortar, and the reduction was more significant as the volume replacement increased. Zhu Pinghua et al. [31] studied the RFA and mortar’s ITZ in geopolymer mortar, and found that under alkaline conditions, on the surface of recycled fine aggregate, a relatively dense ITZ was formed by reacting the old mortar with the new slurry, which mitigated the adverse effects of RFA on geopolymer mortar.

Although researchers have observed the influence of RFA on geopolymer mortar at different replacement levels in existing research and obtained conclusions that RFA incorporation can adversely affect mechanical properties, they have not determined the reasonable proportion of RFA to be used in geopolymers. There are still contradictions regarding the strength of the connection between the RFA surface and the geopolymer slurry, the variety of RFA in the studies is relatively similar, and RFA of varying quality has not been used for comparison. Furthermore, the RFA preprocessing method, which is very effective in concrete, has not been applied in geopolymer mortars. Therefore, it is important to systematically investigate the application of different levels of RFA in geopolymer mortars and to verify whether the preprocessing method of RFA in geopolymer mortars is still effective.

In this experiment, GGBS and FA were used as raw materials to produce geopolymer mortar, and different degrees (0%, 25%, 50%, 75%, 100%) of NA were replaced by RFA. The RFA used was of two qualities: Class M (RSM) and Class L (RSL), and RSL2 and RSM2 after the RSM and RSL had been sieved to remove fine particles (<0.15 mm). In this study, we tested the geopolymer mortar’s fresh properties, mechanical properties, and drying shrinkage, and observed and summarized the effects of different qualities of RFA and optimized RFA on geopolymer mortars. We then characterized the ITZ changes between RFA and geopolymer paste through microstructural observations, and the relationship between the number of fine pores and compressive strength. The study also explored whether the removal of particles below 0.15 mm in RFA could improve the performance of RFA in geopolymer mortars. These findings could contribute to the broader application of RFA in geopolymer mortar.

## 2. Materials and Experimental Programs

### 2.1. Materials

Table 1 shows the physical properties of the FA, GGBS, and solution. As binders and aluminosilicate material, FA and GGBS conforming to JIS A 6201 [32] and JIS A 6206 [33] were used. FA was from the power plant in Reihoku of Kumamoto in Japan. Table 2 provides the chemical compositions of both the fly ash and GGBS. To prepare the alkaline activator (AL) for this study, NaOH and Na_2_SiO_3_ were mixed. The NaOH solution, with approximately 98% weight NaOH, was prepared in distilled water at a concentration of 12 mol/L. The NaOH solution was left for 24 h before mixing. In the Na_2_SiO_3_ fluid, the content of SiO_2_, Na_2_O, and water was 29.4%, 14.7%, and 55.9%. In AL, the weight ratio of Na_2_SiO_3_/NaOH was 2.0 based on the studies by Muhammad N.S. Hadi [34].

Sea sand (S) from Kitakyushu Iwaya in Japan, recycled fine aggregate (RSM, RSL) conforming to the Class M and L standards of JIS A 5022 [35], and treated recycled fine aggregate (RSM2, RSL2) were used as fine aggregates in this experiment. The standard of Class M and Class L represented the different qualities of RFA. The aggregate quality of Class M was higher than that of Class L, indicating its suitability for use in structural concrete. Recycled aggregate L is primarily produced by crushing concrete chunks, which are generated from the demolition of concrete structures, using crushers. It is an aggregate for concrete that has not undergone advanced processing. In other words, Class M is the middle quality and Class L is the low quality. The specifications of RSM and RSL are shown in Table 3.

**Figure 1:**
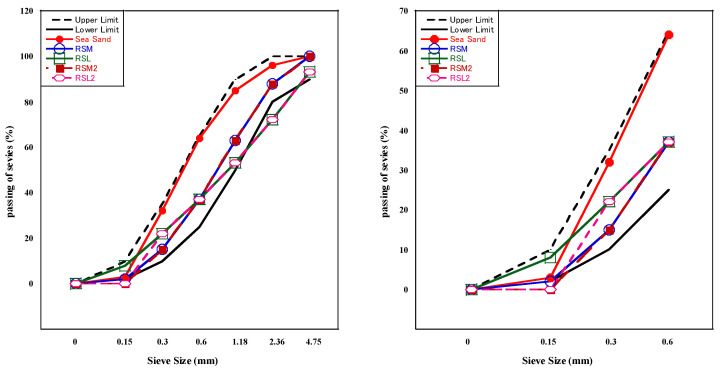
Particle size distribution and enlarged view.

RSM2 and RSL2 were produced from RSM and RSL through a processing procedure, which manually removed some of the fine powder particles (<0.15 mm) in the RFA to improve its properties. The fine aggregate’s physical properties are shown in Table 4. Absolute dry density refers to the mass of sand per unit volume in a completely dry state; that is, without any moisture. On the other hand, surface dry density refers to the density of sand in a saturated surface-dry condition. In this state, all the pores of the sand are filled with water, but there is no excess water on the surface. Given that the RFA used in this paper had high water absorption rates, to ensure the accuracy of each mix proportion and prevent the absorption of water from the solution, the RFA used in these experiments was in a surface-dry state. The fineness modulus of sand is an index used to measure the aggregate particle size distribution. A larger fineness modulus indicates coarser aggregate; a smaller fineness modulus indicates finer aggregate. The particle size distribution of fine aggregate is shown in Figure 1.

### 2.2. Mixing and Preparing the Specimens

Table 5 shows the mix proportions. The unit alkaline activator amount was 388 kg/m^3^; alkaline activator/powder (AL/P) of 0.6 by weight was used. N mortar prepared with sea sand served as the control group. RFA replaced sea sand by volume at 25%, 50%, 75%, and 100%.

We used a 5 L mortar mixer to mix the geopolymer mortar, added FA and fine aggregates to the blender and blended for 1 min. Then, we added AL and mixed for 5 min, waited 15 s, then mixed for 1 min. The mold was filled with geopolymer mortar and vibrated for 30 s. The curing method was based on Narayanan’s study [36]. The mold was placed in a sealed bag and cured at 80 °C for 6 h. Then, we demolded the sample and put it into the laboratory environment to continue curing. The laboratory environment was 20 ± 1.0 °C and an RH of 60 ± 5%.

### 2.3. Testing

The test items include flowability, compressive strength, drying shrinkage, SEM-EDS, and porosity analysis. According to JIS R 5201 [37] and JIS A 1108 [38], the compressive strength of mortar were tested, and the dimension of specimens was 50 mm × 100 mm. In 3, 7, and 28 days we tested the compressive strength.

**Figure 2:**
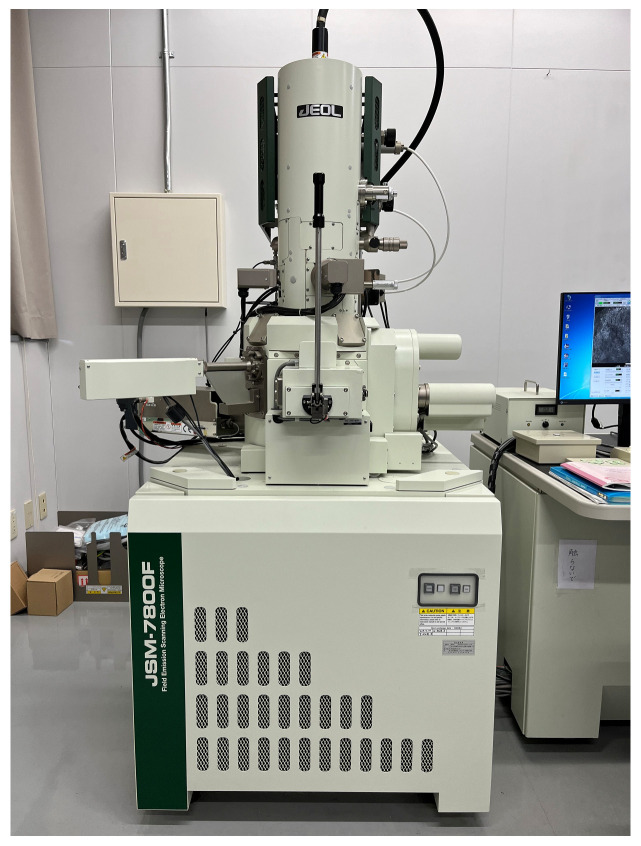
Scanning electron microscope.

**Figure 3:**
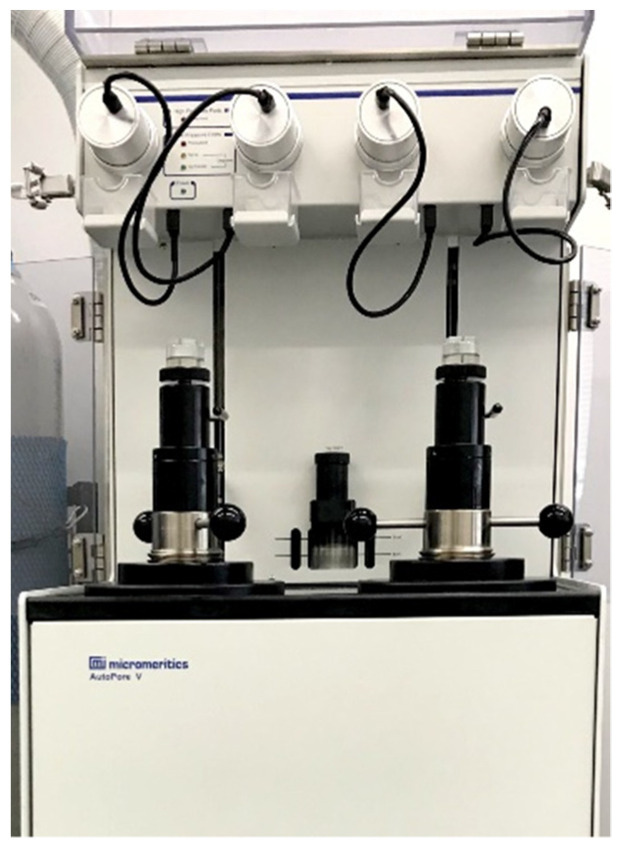
AutoPore V.

The drying shrinkage experiment was performed based on JIS A 1129-2 [39], and prismatic specimens measuring 40 × 40 × 160 mm were produced. For SEM-EDS measurement, small cube-shaped mortar samples were used, placed in a specific mold, and then treated with resin. The analyzing instrument was a Zeiss field emission scanning electron microscope; the SEM-EDS equipment is shown in Figure 2. For the porosity measurement, a laboratory-cured specimen (φ50 × 100 mm) was crushed and sieved to create a particle group of 2.5 to 5.0 mm. We immersed the sample in acetone to stop the geopolymerization and vacuum dried it for 72 h before use. The porosity was measured on day 7. The measurement was performed using mercury intrusion porosimetry (MIP); the MIP equipment is shown in Figure 3.

## 3. Result and Discussion

### 3.1. Fresh Properties

The flowability of mortar refers to its ability to flow during construction and is an important performance indicator of mortar. The flowability of mortar can affect its properties, such as drying shrinkage, permeability, and crack resistance [40,41]. Therefore, researching the flowability of mortar is of great importance.

**Figure 4:**
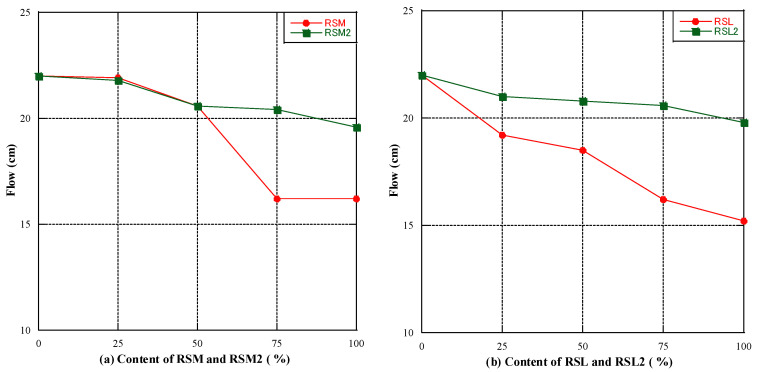
The flowability (**a**) RSM and RSM2 used as RFA, (**b**) RSL and RSL2 used as RFA.

The flowability of geopolymer mortar with RSM and RSM2 added is shown in Figure 4a, while Figure 4b shows the same with RSL and RSL2 added. As shown in Figure 4, it can be observed that with the increase of RFA content, the flowability of mortar decreased and reached its lowest when the content of RFA was 100%. The results show that the flowability of mortar decreased with an increasing RFA replacement rate, irrespective of the RFA quality. Butler et al. [42] reported that natural sand mortar had better flowability than recycled aggregate mortar. Comparing Figure 4a and 4b, irrespective of whether considering RSM and RSL or RSM2 and RSL2, the flowability of class M outperformed that of class L. This could be because the superior quality of Class M RFA might have had particles with more regular shapes and smoother surfaces, leading to reduced internal friction. The article by S.K. [43] indicates that the rougher the surface texture and the more angular the edges of RFA are, the greater the internal friction it imparts to the mortar, thereby affecting the mortar’s workability.

For an equivalent RFA content, the flowability of both RSM2 and RSL2 surpassed that of RSM and RSL. This signifies that the removal of particles smaller than 0.15 mm from RFA effectively enhanced the flowability of geopolymer mortar. Fine particles might have acted as “fillers” in the mortar, increasing friction and consequently affecting flowability. The pre-treatment process, by removing these fine particles, enhanced the mortar’s flowability.

### 3.2. Compressive Strength

**Figure 5:**
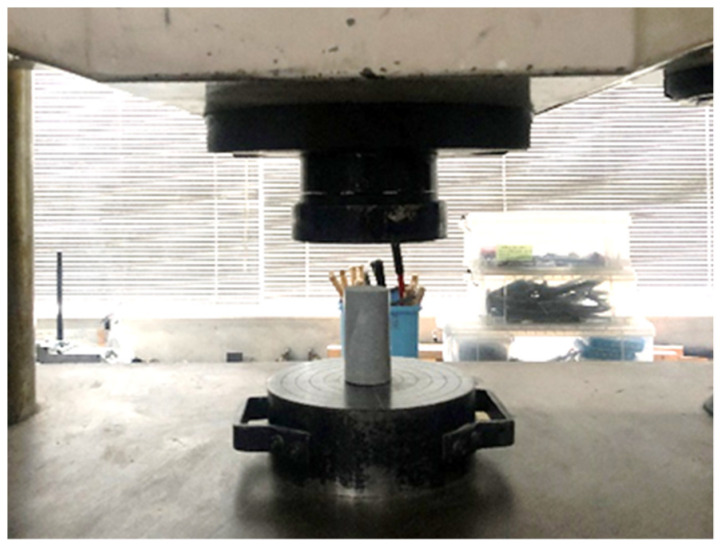
The testing of compressive strength (200 kN capacity).

**Figure 6:**
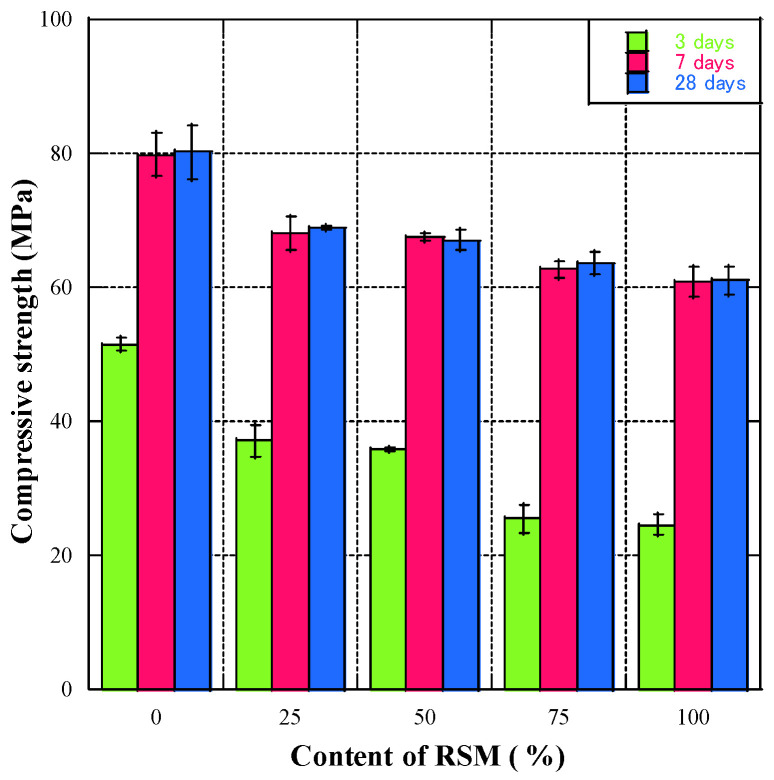
The compressive strength (RSM).

The testing of compressive strength is shown in Figure 5. Figure 6 shows how the compressive strength of geopolymer mortar varied with curing age and RSM replacement rate. The results show that when the curing time was from 3 to 7 days, the compressive strength of geopolymer mortar increased greatly. However, the compressive strength of the geopolymer mortar almost remained unchanged at a curing age from 7 to 28 days. This may be due to initial high-temperature curing: high-temperature curing at 80 °C accelerated the reaction between the geopolymer and solution, leading to a marked compressive strength increase in the early period (3d, 7d) [44]. Görhan [45] reported that the compressive strength of geopolymer mortar reached its maximum value on the 7th day when high-temperature curing was applied. Hence, the geopolymer mortar’s compressive strength growth under high-temperature curing occurred mainly in the early stage and peaked at around 7 days.

**Figure 7:**
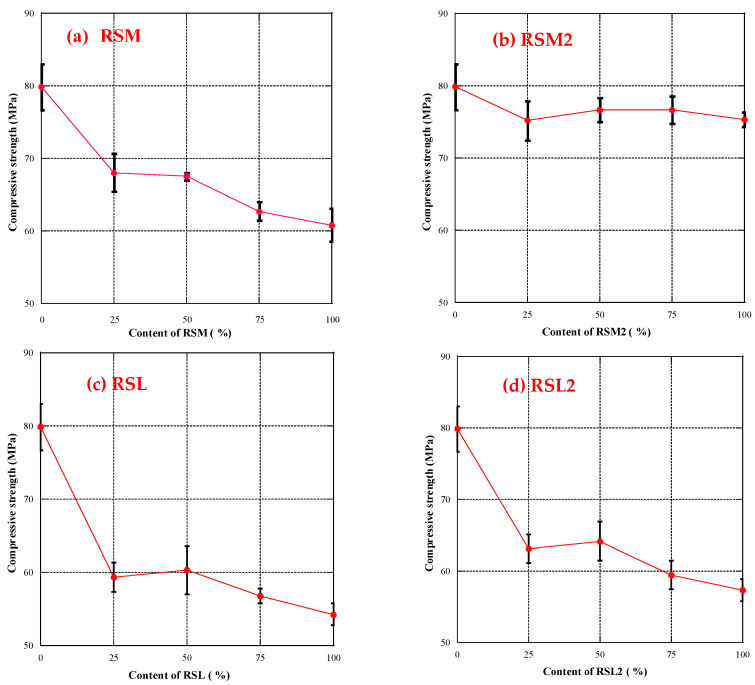
The compressive strength (**a**) RSM, (**b**) RSM2, (**c**) RSL, and (**d**) RSL2.

In Figure 7a,c, it can be observed that when RSM and RSL were incorporated as an RFA into geopolymer mortar, with the change of RFA dosage, the compressive strength of RGM showed a trend of first decreasing, then remaining almost unchanged, and then decreasing again. When the substitution rate of RFA was 25%, the compressive strength of RGM was significantly reduced compared with that of the N group. When the substitution rate of RFA was 50%, the compressive strength of RGM did not decrease compared to the RGM with a substitution rate of 25%. However, when the substitution rate of RFA exceeded 50%, the compressive strength of RGM decreased again until the substitution rate of RFA reached 100%.

There are two reasons for the significant variation in the compressive strength of the mortar. One is that the material properties of RFA are relatively poor compared to natural aggregates [46], so even when the substitution rate of RFA is only 25%, it still significantly lowers the mortar’s compressive strength. On the other hand, a secondary reaction occurs under alkaline stimulation due to the old slurry and unreacted silica and alumina on the RFA surface forming a relatively strong transition zone between the recycled aggregate and the new geopolymer slurry. This partially compensates for the poor material properties of RFA [31,47,48]. Hence, the mortar’s compressive strength does not drop much when the RFA replacement rate increases from 25% to 50%, and even at 100% replacement, the compressive strength is not much lower than at 25% replacement (with a strength difference of only 8 MPa and 5 MPa).

When using the same dosage, the mortar’s compressive strength with RSL is much lower than with RSM. This is because the sand quality in the mortar significantly affects its mechanical properties [49], and according to the description under JIS A 5022 [35], the quality of RSL is lower than that of RSM. Based on the above reasons, this results in lower compressive strength of mortar using RSL than RSM.

Figure 7b,d displays the compressive strength of RGM using RSM2 and RSL2. From the figure, it can be observed that when RSM2 replaced NA, the compressive strength decreased, but unlike when using RSM, the compressive strength did not decrease very significantly even when the RSM2 dosage was changed. Therefore, it can be inferred that the dosage of RSM2 does not considerably impact compressive strength. Furthermore, when comparing the RSM and RSM2 at the same dosage, it is found that the geopolymer mortar with added RSM2 is higher than that with added RSM, suggesting that removing some of the fine powder particles in RSM2 improved its material performance and reduced the adverse impact on the compressive strength of geopolymer mortar.

Adding RSL2 to RGM affected its compressive strength similarly to adding RSL. The compressive strength first dropped then stayed constant, and then dropped again as the RSL2 content increased. The compressive strength reached the lowest when 100% RSL2 was added. Therefore, it can be understood that compared to using NA, the addition of RSL2 will reduce the compressive strength. Comparing the use of RSL in geopolymer mortar, it is found that when the same amount of RFA is added, the compressive strength of RGM using RSL2 is higher than that using RSL. This indicates that reducing the content of fine powder particles in RSL can effectively reduce the detrimental impact of RFA on the mortar’s compressive strength.

When using processed RFA (RSM2 and RSL2), the higher the inclusion level (above 75%), the more pronounced was the advantage in enhancing the compressive strength compared to unprocessed RFA. This further underscores the importance of processing RFA to remove fine powder particles of small diameter (<0.15 mm), especially at high inclusion levels.

Both the inclusion amount and quality of RFA affected the compressive strength of geopolymer mortar. The addition of a certain amount of RFA led to a decline in compressive strength. RSM exhibited better performance in RGM compared to RSL. Processing RFA, by removing some fine particles of small diameter, effectively improved its performance in geopolymer mortar.

### 3.3. Drying Shrinkage

The drying shrinkage of mortar refers to the phenomenon that the volume of mortar changes during the gradual drying process due to the evaporation of water. Specifically, as the surface water of the mortar evaporates, internal water migrates to the surface through capillary pores, resulting in a decrease in pore structure and subsequent shrinkage deformation of the mortar [50,51]. Mortar’s drying shrinkage can affect the stability and appearance of buildings, particularly in special situations such as high-rise buildings and long tunnels, where the accumulation of small displacements caused by drying shrinkage can lead to structural instability [52,53,54]. Therefore, the study of drying shrinkage of mortar and its control methods is of great significance to the safety and durability of buildings.

**Figure 8:**
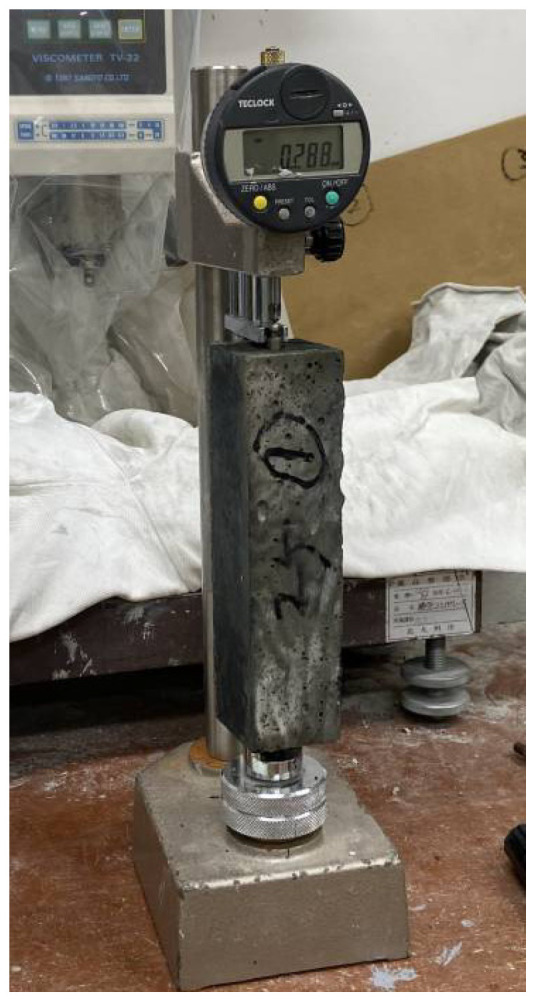
Testing drying shrinkage.

**Figure 9:**
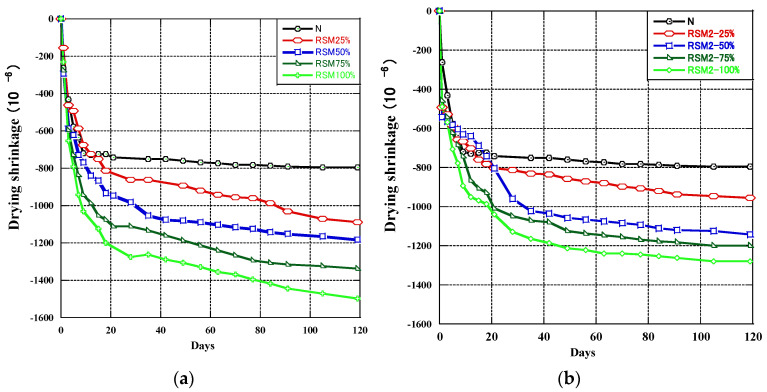
The drying shrinkage of (**a**) RSM, (**b**) RSM2.

Figure 8 shows the testing of drying shrinkage. The drying shrinkage of geopolymer mortar using RSM and RSM2 as recycled aggregates is shown in Figure 9. Figure 9a depicts the drying shrinkage when RSM was used as RFA. It can be observed that the drying shrinkage depended on the RSM content. Specifically, as the content of RSM increased, the drying shrinkage of geopolymer mortar also increased, and the maximum drying shrinkage was reached when the content of RSM was 100%. Before the curing age of 28 days, the drying shrinkage of the mortar increased rapidly. After the curing age of 28 days, the drying shrinkage rate of group N mortar slowed down and gradually decreased with the increase of the curing period until it was nearly zero. The drying shrinkage rate of mortar using RSM also slowed down, but unlike group N mortar, it still showed a certain drying shrinkage rate after 100 days.

Figure 9b shows the drying shrinkage when RSM2 was recycled aggregates. The drying shrinkage was influenced by RSM2 content, increasing as the RSM2 content increased and reaching its maximum value at 100% RSM2 content. When the curing age is divided into segments, the drying shrinkage rate of geopolymer mortar was high during the first 20 days of curing. Still, it gradually decreased after 20 days until it almost stopped shrinking.

Based on the above results, using RFA as a replacement for NA in geopolymer mortar has a negative impact. This is similar to the situation where RFA is used in cement [55,56,57]; the addition of RFA increases the drying shrinkage compared with nature sand. The use of RFA increases the rate and value of dry shrinkage of geopolymer mortar, and the negative impact becomes more significant as the replacement rate of RFA increases.

However, the impact of RFA use on the drying shrinkage of cement mortar and geopolymers mortar varies. For example, Wu’s [58] study indicates that when RFA content in cement mortar is 25%, 50%, and 100%, the 28 days drying shrinkage of the cement mortar increases by 4.5%, 10.6%, and 38.0%, respectively, compared to the original mortar without RFA. In this experiment, when RSM content in geopolymer mortar was 25%, 50%, and 100%, the 28 days drying shrinkage of the geopolymer mortar increased by 20%, 37%, and 78%, respectively, compared to the original mortar without RSM. The significant difference between the two suggests that compared to cement mortar, the use of RFA has a more significant impact on the drying shrinkage of geopolymer mortar.

By comparing the dry shrinkage of geopolymer mortar using RSM and RSM2, it was found that removing fine powder particles can reduce its negative impact on the dry shrinkage of geopolymer mortar. This not only reduced the dry shrinkage value of geopolymer mortar, but also lowered its dry shrinkage rate during long-term curing.

This signifies that the preprocessing method, which involves the removal of fine powder particles (<0.15 mm), indeed enhances the performance of RFA in geopolymer mortar. Particularly at higher replacement levels, RSM2 demonstrated superior performance compared to RSM, indicating that preprocessing can effectively mitigate the adverse effects introduced by RFA.

### 3.4. SEM

**Figure 10:**
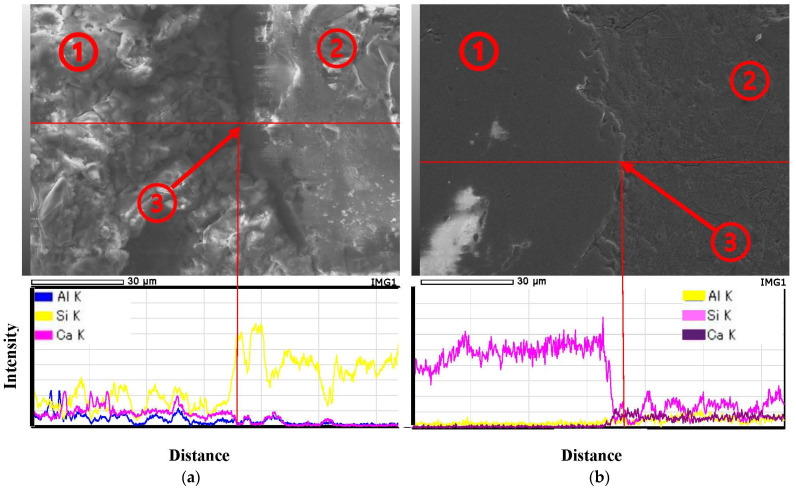
The SEM and EDS (**a**) the ITZ of NA, (**b**) the ITZ of RFA.

As is well known, in recycled concrete and mortar, the interfacial transition zone (ITZ) is the weakest part between the aggregate and paste, and microcracks usually first develop under loading. Further investigation of these areas is crucial because the ITZ has a unique microstructure compared to most adjacent pastes [58,59,60]. Figure 10a is a micrograph of a geopolymer mortar without RFA, with positions marked by white lines for energy spectrum analysis. The corresponding energy spectrum graphs and element contents are shown below the image. Based on the element content graph, it can be seen that the Si element mass percentage in Region 2 is very high, and the Ca element mass percentage is meager, suggesting that Region 2 is natural sand. Region 1 has a Si mass percentage of around 20% and Al and Ca mass percentages of around 10%, indicating that it is a product of geopolymerization, the geopolymer paste. Region 3 is the transition zone between mortar paste and natural sand. In cement mortar, the fine aggregate and paste connection is the most fragile, and microcracks are easily generated under loading. The study of Liu et al. [61] shows that the microcracks in ITZs between NA and hardened mortar first start under loading. There are also significant cracks in the ITZ between natural sand and the paste, a conclusion that still applies to geopolymer mortar.

The microstructure of the ITZ in geopolymer mortar with 100% content of RFA is shown in the right micrograph. Based on the element mass powder analysis, it can be inferred that Region 2 is recycled sand, Region 1 is geopolymer binder, and Region 3 is ITZ. Observations from the micrograph reveal that the ITZ region is very dense, and the connection between the aggregate and the binder is very tight, with no apparent cracks. This is attributed to the fact that the surface of the RFA is attached to old cement paste, which can react again under alkaline conditions. With the increase of the curing age, these old and new pastes will bond well together, and the boundary of the ITZ will no longer be obvious [62,63,64].

Comparing the ITZ of RS-GP (natural sand and binder) and RFA-GP (recycled fine aggregate and binder) reveals that RFA can result in a more compact ITZ than natural sand, which is one of the reasons why the compressive strength does not significantly decrease even when the RFA’s replacement rate reaches 100%. Moreover, microcracks in the ITZ can cause a high porosity of the mortar, making it easier for harmful substances such as chlorides, carbon dioxide, and sulfates to penetrate into the internal structure of the mortar, thereby affecting the durability of both the mortar and concrete [58]. In this experiment, due to the denser nature of the ITZ in RFA-GP, although the drying shrinkage increases with the increase of RFA substitution rate, this disadvantage can be alleviated by generating a dense ITZ, thus increasing the applicability of RFA.

### 3.5. Correlation of Porosity and Compressive Strength of the Recycled Geopolymer Mortar

As is well known, fine pore size is an essential experimental parameter when studying concrete and mortar. Fine pore size refers to the size of tiny pores within the mortar, typically ranging from a few microns to several tens of microns. Fine pore size can affect the mechanical properties of concrete and mortar, mainly compressive and tensile strength. The internal structure and morphology of the mortar can be revealed by studying fine pore size, which is a critical component of mortar. This can help to understand the physical and mechanical properties of mortar better.

**Figure 11:**
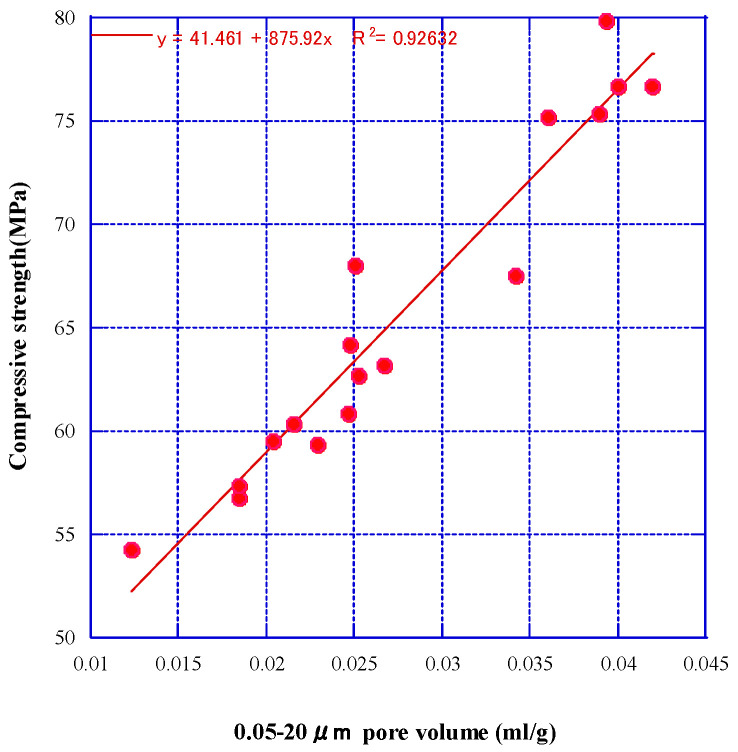
Correlation between 0.05–20 μm cumulative pore volume and compressive strength.

Figure 11 depicts the relationship between the compressive strength of mortar using RSM, RSM2, RSL, and RSL2 and the cumulative pore volume of fine pore sizes ranging from 0.05 μm to 20 μm.

It can be observed that within this range of pore volumes, there is a strong correlation between cumulative pore volume and compressive strength of mortar when using RSM, RSM2, RSL, and RSL2. However, in contrast to the inverse relationship between compressive strength and pore volume in cement mortar, in this experiment, cumulative pore volume in the 0.05–20 μm range correlated positively with mortar strength. This finding conflicts with the conclusion that as the pore volume decreases, concrete or mortar’s compressive and tensile strength also increase. According to Liu, pore structure affects compressive strength. Pores larger than 0.05 μm are harmful and reduce strength [65].

However, as revealed in the SEM analysis in the previous chapter, the main reason for this result is that compared with the clearly visible ITZ with microcracks between natural sand and geopolymer mortar, a dense ITZ can be formed between recycled aggregate and geopolymer paste, which leads to differences in pore volume. For example, in the case of using 100% natural sand, the presence of microcracks between natural sand and geopolymer slurry causes a high pore volume to be obtained. Kuri’s research also showed that a weak ITZ increased porosity [66]. As the amount of RFA increases, the ITZ between natural sand with a high pore volume and paste decreases and is replaced by a dense ITZ between RFA and paste. This reduces the pore volume of the mortar, and there exists a certain positive correlation between the compressive strength and the substitution rate of RFA. Therefore, the relationship equation shown in Figure 11 can be obtained.

## 4. Conclusions

This study examined how RFA quality affects FA/GBBS-based geopolymer concrete in fresh and hardened states. The main findings and suggestions are:

1. RFA distinctly affects the flowability of geopolymer mortar. Opting for a higher-quality RFA, such as Class M, and administering appropriate preprocessing of RFA, such as by eliminating particles below 0.15 mm, can efficaciously augment the flowability of the mortar.
2. The compressive strength growth of RGM is concentrated in the early curing stage. The addition of a certain amount of RFA leads to a decline in strength. The quality of the sand in the mortar has a great impact on the mechanical properties of the mortar. In the case of using the same dosage, the compressive strength of mortar using RSL is significantly lower than that of using RSM.
3. Processing RFA by removing some fine particles of small diameter can effectively improve its performance in geopolymer mortar. The higher the inclusion level (above 75%), the more pronounced is the advantage in enhancing the compressive strength compared to unprocessed RFA.
4. RFA adversely affects the drying shrinkage of geopolymer mortar. However, through proper processing, such as the removal of fine particles, its detrimental impact on the mortar’s drying shrinkage can be significantly mitigated.
5. In RGM, RFA can result in a more compact ITZ than NA, which is one of the reasons why the compressive strength does not significantly decrease when the dosage of RFA increase.
6. There is a positive correlation between the pore volume and the compressive strength of geopolymer mortar using RFA, in contrast to the conclusion in cement mortar.

## Figures and Tables

**Table 1:** Materials.

Alkaline activator	Distilled water		AL
	Sodium hydroxide (NaOH)	The concentrations of 12 M	
	Sodium silicate (Na_2_SiO_3_)	12.97% Na_2_O, 29.03% SiO_2_ and 58% H_2_O	
Admixture	Fly ash (adapted to JISⅡ)	Density 2.30 (g/cm^3^), LOI 1.3% specific surface area 4000 (g/cm^3^)	FA
	Ground granulated blast-furnace slag (adapted to JIS)	Density 2.91 (g/cm^3^), LOI 0.04% specific surface area 4100 (g/cm^3^)	GGBS

**Table 2:** Chemical composition of the fly ash in this study.

	SiO_2_ (%)	Al_2_O_3_ (%)	Fe_2_O_3_ (%)	CaO (%)	SO_3_ (%)	MgO (%)
Fly ash	53.8	13.5	13	8.99	0.49	1.48
GGBS	32.7	13.4	0.5	41.6	6.9	0.3

**Table 3:** Specifications of recycled aggregates Class M and Class L.

Type	Symbol	Summary	Range of Particle Size (mm)	Absolute Dry Density (g/cm^3^)	Water Absorption (%)	Fine Particle Content (%)
Class M	RSM	Fine aggregate processed by crushing and grinding the original concrete, with particle size distribution adjusted as necessary.	<5	>2.2	<7	<8
Class L	RSL	Fine aggregate produced by processing the original concrete through crushing	<5	-	<13	<10

**Table 4:** Properties of the fine aggregates.

Properties	S	RSM	REM2	RSL	RSL2	JIS A5022(M)	JIS A5022(L)
Absolute dry density (g/cm^3^)	2.64	2.29	2.25	2.09	2.08	>2.3	-
Fineness modulus	2.2	3.09	3.09	3.15	2.2	-	-
Water absorption (%)	1.06	6.98	6.35	9.91	9.79	<7.0	<13.0
Saturated surface dry density (g/cm^3^)	2.67	2.46	2.402	2.32	2.30		

**Table 5:** Mix proportion.

Type					Unit Mass (kg/m^3^)				
	AL/P	AL	FA	GGBS	S	RSM	RSM2	RSL	RSL2
N	0.60	388	162	492	1322	0	0	0	0
RSM-25	0.60	388	162	492	992	287	0	0	0
RSM-50	0.60	388	162	492	661	574	0	0	0
RSM-75	0.60	388	162	492	331	860	0	0	0
RSM-100	0.60	388	162	492	0	1147	0	0	0
RSM2-25	0.60	388	162	492	992	0	282	0	0
RSM2-50	0.60	388	162	492	661	0	564	0	0
RSM2-75	0.60	388	162	492	331	0	845	0	0
RSM2-100	0.60	388	162	492	0	0	1127	0	0
RSL-25	0.60	388	162	492	992	0	0	262	0
RSL-50	0.60	388	162	492	661	0	0	524	0
RSL-75	0.60	388	162	492	331	0	0	785	0
RSL-100	0.60	388	162	492	0	0	0	1047	0
RSL2-25	0.60	388	162	492	992	0	0	0	261
RSL2-50	0.60	388	162	492	661	0	0	0	521
RSL2-75	0.60	388	162	492	331	0	0	0	782
RSL2-100	0.60	388	162	492	0	0	0	0	1042

## Data Availability

The data presented in this study are available on request from the corresponding author. The data are not publicly available due to privacy restrictions.

## References

[1] Behera M., Bhattacharyya S.K., Minocha A.K., Deoliya R., Maiti S. (2014). Recycled aggregate from C&D waste & its use in concrete–A breakthrough towards sustainability in construction sector: A review. Constr. Build. Mater..

[2] Global Energy Review 2021, Global Energy Review 2021—Analysis—IEA. https://iea.blob.core.windows.net/assets/d0031107-401d-4a2f-a48b-9eed19457335/GlobalEnergyReview2021.pdf.

[3] Freedonia Group World construction aggregates. https://cdn.cocodoc.com/cocodoc-form-pdf/pdf/58304896--World-Construction-.pdf.

[4] Evangelista L., Guedes M., de Brito J., Ferro A., Pereira M. (2015). Physical, chemical and mineralogical properties of fine recycled aggregates made from concrete waste. Constr. Build. Mater..

[5] Kim J. (2022). Influence of quality of recycled aggregates on the mechanical properties of recycled aggregate concretes: An overview. Constr. Build. Mater..

[6] Peng C.-L., Grosskopf K.R., Kibert C.J. Construction Waste Management and Recycling Strategies in the United States. Proceedings of the First Conference of CIB TG 16 on Sustainable Construction.

[7] Lu W., Webster C., Chen K., Zhang X., Chen X. (2017). Computational Building Information Modelling for construction waste management: Moving from rhetoric to reality. Renew. Sustain. Energy Rev..

[8] Klepa R.B., Medeiros M.F., Franco M.A.C., Tamberg E.T., Farias T.M.D.B., Paschoalin Filho J.A., Berssaneti F.T., Santana J.C.C. (2019). Reuse of construction waste to produce thermoluminescent sensor for use in highway traffic control. J. Clean. Prod..

[9] Liu B., Feng C., Deng Z. (2019). Shear behavior of three types of recycled aggregate concrete. Constr. Build. Mater..

[10] Kumar G.S. (2019). Influence of fluidity on mechanical and permeation performances of recycled aggregate mortar. Constr. Build. Mater..

[11] Kanadasan J., Razak H.A. (2015). Utilization of palm oil clinker as cement replacement material. Materials.

[12] Karim M.R., Hashim H., Abdul Razak H., Yusoff S. (2017). Characterization of palm oil clinker powder for utilization in cement-based applications. Constr. Build. Mater..

[13] Turner L.K., Collins F.G. (2013). Carbon dioxide equivalent (CO_2_-e) emissions: A comparison between geopolymer and OPC cement concrete. Constr. Build. Mater..

[14] Kumar M.L., Revathi V. (2020). Microstructural Properties of Alkali-Activated Metakaolin and Bottom Ash Geopolymer. Arab. J. Sci. Eng..

[15] Imtiaz L., Rehman S.K.U., Memon S.A., Khan M.K., Javed F. (2020). A review of recent developments and advances in eco-friendly geopolymer concrete. Appl. Sci..

[16] Haddad R.H., Alshbuol O. (2016). Production of geopolymer concrete using natural pozzolan: A parametric study. Constr. Build. Mater..

[17] Albidah A., Alqarni A.S., Abbas H., Almusallam T., Al-Salloum Y. (2022). Behavior of Metakaolin-Based geopolymer concrete at ambient and elevated temperatures. Constr. Build. Mater..

[18] Dapena E., Alaejos P., Lobet A., Pérez D. (2011). Effect of recycled sand content on characteristics of mortars and concretes. J. Mater. Civ. Eng..

[19] Zhao Z., Remond S., Damidot D., Xu W. (2015). Influence of fine recycled concrete aggregates on the properties of mortars. Constr. Build. Mater..

[20] Braga M., De Brito J., Veiga R. (2012). Incorporation of fine concrete aggregates in mortars. Constr. Build. Mater..

[21] Neno C., Brito J., Veiga R. (2014). Using fine recycled concrete aggregate for mortar production. Mater. Res..

[22] Katz A. (2004). Treatments for the improvement of recycled aggregate. J. Mater. Civ. Eng..

[23] Luo M., Dai J., Ding Z., Liu Y. (2022). Properties of Mortar Containing Recycled Fine Aggregate Modified by Microbial Mineralization. Buildings.

[24] Tateyashiki H., Shima H., Matsumoto Y., Koga Y. (2001). Properties of concrete with high quality recycled aggregate by heat and rubbing method. Proc. JCI.

[25] Tam V.W.Y., Tam C.M., Le K.N. (2007). Removal of cement mortar remains from recycled aggregate using pre-soaking approaches. Resour. Conserv. Recycl..

[26] Zhang J., Shi C., Li Y., Pan X., Poon C.-S., Xie Z. (2015). Influence of carbonated recycled concrete aggregate on properties of cement mortar. Constr. Build. Mater..

[27] Li B., Hou S., Duan Z., Li L., Guo W. (2021). Rheological behavior and compressive strength of concrete made with recycled fine aggregate of different size range. Constr. Build. Mater..

[28] Saba M., Assaad J.J. (2021). Effect of recycled fine aggregates on performance of geopolymer masonry mortars. Constr. Build. Mater..

[29] Hasnaoui A., Ghorbel E., Wardeh G. (2021). Performance of metakaolin/slag-based geopolymer concrete made with recycled fine and coarse aggregates. J. Build. Eng..

[30] Lyu B.-C., Guo L.-P., Fei X.-P., Wu J.-D., Bian R.-S. (2023). Preparation and properties of green high ductility geopolymer composites incorporating recycled fine brick aggregate. Cem. Concr. Compos..

[31] Zhu P., Hua M., Liu H., Wang X., Chen C. (2020). Interfacial evaluation of geopolymer mortar prepared with recycled geopolymer fine aggregates. Constr. Build. Mater..

[32] (2015). Fly Ash for Use in Concrete.

[33] (2013). Ground Granulated Blast-Furnace Slag for Concrete.

[34] Hadi M.N.S., Al-Azzawi M., Yu T. (2018). Effects of fly ash characteristics and alkaline activator components on compressive strength of fly ash-based geopolymer mortar. Constr. Build. Mater..

[35] (2018). Recycled Aggregate Concrete-Class M.

[36] Narayanan A., Shanmugasundaram P. (2017). An experimental investigation on flyash-based geopolymer mortar under different curing regime for thermal analysis. Energy Build..

[37] (2013). Physical Testing Methods for Cement.

[38] (2018). Method of Test for Compressive Strength of Concrete and Mortar.

[39] (2010). Method of Measurement for Length Change of Mortar and Concrete—Part 2: Method with Contact-Type Strain Gauge.

[40] Hassan I.O., Ismail M., Forouzani P., Majid Z.A., Mirza J. (2014). Flow characteristics of ternary blended self-consolidating cement mortars incorporating palm oil fuel ash and pulverised burnt clay. Constr. Build. Mater..

[41] Lachemi M., Hossain K.M.A., Patel R., Shehata M., Bouzoubaâ N. (2007). Influence of paste/mortar rheology on the flow characteristics of high-volume fly ash self-consolidating concrete. Mag. Concr. Res..

[42] Butler L.J., West J.S., Tighe S.L. (2014). Towards the classification of recycled concrete aggregates: Influence of fundamental aggregate properties on recycled concrete performance. J. Sustain. Cement-Based Mater..

[43] Kirthika S.K., Singh S., Chourasia A. (2020). Alternative fine aggregates in production of sustainable concrete—A review. J. Clean. Prod..

[44] De Vargas A.S., Dal Molin D.C., Vilela A.C., Silva F.J.D., Pavão B., Veit H. (2011). The effects of Na_2_O/SiO_2_ molar ratio, curing temperature and age on compressive strength, morphology and microstructure of alkali-activated fly ash-based geopolymers. Cement Concr. Compos..

[45] Görhan G., Kürklü G. (2014). The influence of the NaOH solution on the properties of the fly ash-based geopolymer mortar cured at different temperatures. Compos. Part B Eng..

[46] Behera M., Minocha A.K., Bhattacharyya S.K. (2019). Flow behavior, microstructure, strength and shrinkage properties of self-compacting concrete incorporating recycled fine aggregate. Constr. Build. Mater..

[47] Thomas B.S., Yang J., Bahurudeen A., Chinnu S.N., Abdalla J.A., Hawileh R.A., Hamada H.M. (2022). Geopolymer concrete incorporating recycled aggregates: A comprehensive review. Clean. Mater..

[48] Sáez P.V., Osmani M. (2019). A diagnosis of construction and demolition waste generation and recovery practice in the European Union. J. Clean. Prod..

[49] Katz A., Kulisch D. (2017). Performance of mortars containing recycled fine aggregate from construction and demolition waste. Mater. Struct..

[50] Neville A.M. (1995). Properties of Concrete.

[51] Neville A.M., Brooks J.J. (1987). Concrete Technology.

[52] Silva R.V., De Brito J., Dhir R.K. (2015). Prediction of the shrinkage behavior of recycled aggregate concrete: A review. Constr. Build. Mater..

[53] Zhang H., Wang Y., Lehman D.E., Geng Y., Kuder K. (2020). Time-dependent drying shrinkage model for concrete with coarse and fine recycled aggregate. Cement Concr. Compos..

[54] Medjigbodo S., Bendimerad A.Z., Rozière E., Loukili A. (2018). How do recycled concrete aggregates modify the shrinkage and self-healing properties?. Cement Concr. Compos..

[55] Duan Z.H., Poon C.S. (2014). Properties of recycled aggregate concrete made with recycled aggregates with different amounts of old adhered mortars. Mater. Des..

[56] Pedro D.D., De Brito J., Evangelista L. (2017). Structural concrete with simultaneous incorporation of fine and coarse recycled concrete aggregates: Mechanical, durability and long-term properties. Constr. Build. Mater..

[57] Wang Q., Geng Y., Wang Y., Zhang H. (2020). Drying shrinkage model for recycled aggregate concrete accounting for the influence of parent concrete. Eng. Struct..

[58] Wu H., Wang C., Ma Z. (2022). Drying shrinkage, mechanical and transport properties of sustainable mortar with both recycled aggregate and powder from concrete waste. J. Build. Eng..

[59] Mao Y., Liu J., Shi C. (2021). Autogenous shrinkage and drying shrinkage of recycled aggregate concrete: A review. J. Clean. Prod..

[60] Diamond S., Huang J. (2001). The ITZ in concrete—A different view based on image analysis and SEM observations. Cement Concr. Compos..

[61] Liu Q., Xiao J., Sun Z. (2011). Experimental study on the failure mechanism of recycled concrete. Cement Concr. Res..

[62] Colangelo F., Messina F., Cioffi R. (2015). Recycling of MSWI fly ash by means of cementitious double step cold bonding pelletization: Technological assessment for the production of lightweight artificial aggregates. J. Hazard. Mater..

[63] Almadani M., Razak R.A., Abdullah M.M.A.B., Mohamed R. (2022). Geopolymer-based artificial aggregates: A review on methods of producing, properties, and improving techniques. Materials.

[64] Poolman K.D., Kruger D. (2018). International Congress on Polymers in Concrete (ICPIC 2018).

[65] Liu Z., Takasu K., Suyama H., Koyamada H., Liu S., Hao Q. (2022). The Effect of Cementitious Materials on the Engineering Properties and Pore Structure of Concrete with Recycled Fine Aggregate. Materials.

[66] Kuri J.C., Hosan A., Shaikh F.U.A., Biswas W.K. (2023). The Effect of Recycled Waste Glass as a Coarse Aggregate on the Properties of Portland Cement Concrete and Geopolymer Concrete. Buildings.

